# Supplementary material for: Disruption of glutamine transport uncouples the NUPR1 stress-adaptation program and induces prostate cancer radiosensitivity
Source: Cell Commun Signal. 2025 Jul 24;23:351. doi: 10.1186/s12964-025-02344-3 (PMC12291318; doi:10.1186/s12964-025-02344-3)

Supplementary results

Gln starvation and Gln transporter expression in parental (P) and radioresistant (RR) sublines

To clarify whether nutrient deprivation alone drives transporter upregulation, we apply Gln starvation on both P and RR sublines and measured the expression of AATs. In DU145, Gln deprivation significantly increased the expression of *SLC1A5*, *SLC7A5*, *SLC38A1*, and *SLC38A5* in both P and RR cells (Supplementary Figure 5A). Similarly, LNCaP cells, demonstrated increased expression of *SLC1A5*, *SLC7A5*, *SLC38A1*, *SLC38A7*, and *SLC7A7* with more pronounced increases in the RR subline (Supplementary Figure 5B). Additionally, the results of gene expression microarray confirmed significant upregulation of these transporters under Gln starvation (Supplementary Figure 5C). Overall, these findings suggest the activation of adaptive responses to nutrient deprivation.

Phosphoproteomics analysis reveals cell line–specific mechanisms of transporter depletion

To elucidate the molecular mechanisms underlying the effects of transporter depletion, we performed phosphoproteomics analysis on DU145 and LNCaP cells with shRNA-mediated knockdown of *SLC1A5*, *SLC7A5*, and *SLC38A1* (Supplementray Table 5). For each gene of interest, two different shRNAs were used, with the non-specific shRNA plasmid as a control. The cells were transfected with the plasmids listed in Supplementary Table 2 and then selected using puromycin to eliminate untransfected and transiently transfected cells. Knockdown efficiency was confirmed by Western blotting (Supplementary Figure 8A) and qPCR (Supplementary Figure 8B). The phosphoproteomic profiling was performed by Omics Technologies Inc. (USA).

We found that transporter depletion in DU145 cells caused significant alterations in the phosphorylation of the proteins associated with mitochondrial function and apoptosis (Supplementary Table 5). Notably, increased TOMM20 and AIFM1 phosphorylation recapitulate impaired mitochondrial protein import and induction of caspase-independent

apoptosis, respectively [1-5]. TOMM20, Translocase of the outer mitochondrial membrane 20, is essential to import nuclear-encoded proteins into mitochondria [2]. Increased phosphorylation of TOMM20 can alter the efficiency of mitochondrial protein import, leading to mitochondrial dysfunction. Apoptosis-inducing factor, mitochondrion-associated 1 (AIFM1) is involved in caspase-independent apoptosis by inducing chromatin condensation and DNA fragmentation. Increased phosphorylation of AIFM1 may activate caspase-independent apoptosis pathway linked to mitochondrial dysfunction [1-5]. Also, decreased phosphorylation of HSP90AA1 and SIRT1 indicates compromised mitochondrial protein folding and regulation, leading to mitochondrial stress and dysfunction [6, 7]. These alterations are in line with increased basal respiration and proton leak observed in Seahorse assays (Figure 4J).

On the other hand, LNCaP cells place less emphasis on mitochondrial proteins and more on transcription factors and cell cycle regulators (Supplementary Table 5). LNCaP cells bearing transporter knockdown demonstrated augmented phosphorylation of proteins involved in cell cycle regulation and stress responses, such as FOXM1 and HSF1. FOXM1 is a transcription factor critical for cell cycle progression, DNA repair, and genomic stability [8, 9]. Phosphorylation activates FOXM1, promoting the transcription of genes playing a role in G2/M transition and DNA repair. Enhanced FOXM1 activity can lead to cell cycle dysregulation [8, 9]. Phosphorylation activates HSF1, inducing the expression of heat shock proteins that are important for protein folding and protecting against stress [10]. Enhanced activity of these proteins can alter cell cycle progression and upregulation of heat shock proteins, supporting cell survival under stress [8-10]. In addition, knockdown of *SLC1A5*, *SLC7A5*, and *SLC38A1* in LNCaP cells resulted in the upregulation of phosphorylation of the Inner Membrane Mitochondrial Protein (IMMT), which regulates mitochondrial architecture and function [11, 12], HSF1, which is required for the activation of mitochondrial chaperone genes [13], and FOXM1, a recently described OXPHOS regulator [14, 15].

Supplementary material

Supplementary Tables:

Supplementary Table 1. The siRNA sequences used in the study.

Supplementary Table 2. The shRNA expression vectors used in the study.

Supplementary Table 3. The primers used in the study.

Supplementary Table 4. Antibodies used for Western blot analysis.

Supplementary Table 5. A comparative phosphoproteomics analysis of PCa cell lines DU145 and LNCaP with or without knockdown of Gln AATs.

Supplementary Table 6: The upstream regulators identified in PCa cell lines DU145 and LNCaP using Ingenuity Pathway Analysis (IPA) in response to specified treatments.

Supplementary Table 7. A list of 261 *NUPR1* target genes identified using Ingenuity Pathway Analysis (IPA) in the RNA seq datasets for SLC knockdown in DU145 and LNCaP cells.

Supplementary Table 8. Gene Ontology (GO) analysis of the 261-gene list of the *NUPR1* transcriptional targets.

Supplementary Table 9. Gene Ontology (GO) analysis of the 43-gene list of the *NUPR1* transcriptional targets

Supplementary Table 10. Supplementary Table 6. Gene sets used for the correlative analysis.

Supplementary figures:

Supplementary Figure 1. Inhibition of GLS activity reprograms cell metabolism and inhibits tumor growth. (A) Kaplan–Meier survival curves showing the probability of survival for each treatment group over time (days after the start of treatment). The combination therapy group exhibited a trend toward prolonged survival compared to other groups. **(B)** Mouse plasma glutamine (Gln) and glutamate (Glu) levels upon different treatment arms. CB-839-treated groups showed significantly increased Gln levels compared to control (*$p < 0.05$).

Supplementary Figure 2. Overview of multiparametric *in silico* approaches to identify clinically relevant glutamine transporters in prostate cancer. (A) A brief summary of bioinformatics and computational analyses used to select studied amino acid transporters mediating glutamine transport. The approach included evaluating expression levels, functional assays, and clinical relevance in prostate cancer. **(B)** Radiobiological clonogenic survival curves for DU145 and LNCaP, radioresistant (RR) and parental (P) cell sublines cells that are used in the study. Survival fractions were calculated, and curves were fitted using the linear-quadratic model. Error bars represent SD ***$p < 0.001$). **(C)** Correlation of the expression levels of selected Gln transporters with clinical outcomes in the PCa TCGA cohort (n = 498). OS: overall survival; DFS: disease-free survival: BRFS: biochemical recurrence-free survival. **(D)** Heatmap of microarray data showing transporter expression in DU145 and LNCaP P and RR cells. Red color indicates high expression; blue color indicates low expression.

Supplementary Figure 3. Plating efficiency and clonogenic survival in prostate cancer cells upon transporter depletion. (A) qRT-PCR analysis of gene expression in DU145, LNCaP, and PC3 cells upon siRNA-mediated knockdown of indicated genes. Error bars represent SD (*$p < 0.05$, **$p < 0.01$, ***$p < 0.001$). **(B)** Clonogenic survival curves for DU145, LNCaP and PC3 cells upon depletion of indicated transporters and exposure to increasing doses of radiation. Survival fractions were calculated, and curves

were fitted using the linear-quadratic model $S(D)/S(0) = \exp(-\alpha D - \beta D^2)$ using stratified linear regression after natural logarithm transformation. Error bars represent SD. (*p < 0.05, **p<0.01, ***<0.001). **(C)** Bar chart showing plating efficiency (%) of DU145, LNCaP and PC3 cells upon transporter depletion. Error bars represent SD. (*p < 0.05).

Supplementary Figure 4. qRT-PCR analysis of expression of studied transporter and metabolic genes in DU145, LNCaP and PC3 cells upon depletion of *SLC1A5*, *SLC7A5*, and *SLC38A1*. Error bars represent SD (*p < 0.05, **p<0.01, ***<0.001, ****<0.0001).

Supplementary Figure 5. Differential expression of AATs in response to Gln starvation. (A) qRT-PCR analysis of transporter expression in DU145 P and RR cells under glutamine starvation (–Gln) and non-starvation (+Gln) conditions. Expression levels are demonstated as fold change relative to +Gln parental cells. Error bars represent SD (*p < 0.05, ****p < 0.0001). **(B)** qRT-PCR analysis of transporter expression in LNCaP P and RR cells under glutamine starvation (–Gln) and non-starvation (+Gln) conditions. Expression levels are demonstrated as fold change relative to +Gln parental cells. Error bars represent SD (*p < 0.05, ***p < 0.001, ****p < 0.0001). **(C)** Heatmap of microarray gene expression data showing expression of glutamine transporters in DU145 (up) and LNCaP (down) cells under glutamine starvation (–Gln) and non-starvation (+Gln) conditions.

Supplementary Figure 6. Transporter knockdown alters cell cycle distribution and mTORC1 signaling in PCa cells. (A) Cell death analysis in DU145 and LNCaP cells using Annexin V/PI staining following transporter knockdown under sham and irradiated (6 Gy) conditions. Bar charts represent percentages of apoptotic and necrotic cells. Error bars represent SD (*p < 0.05). **(B)** Gene Set Enrichment Analysis (GSEA) for genes significantly up- or down regulated upon *SLC1A5*, *SLC7A5*, *SLC38A1* knockdown revealed that deregulated genes are associated with an inhibition of mTORC1 signaling.

(C) Cell cycle distribution of DU145 and LNCaP cells under sham-irradiated conditions following transporter knockdown, analyzed by flow cytometry. Error bars represent SD (*$p < 0.05$). **(D)** Cell cycle distribution of DU145 and LNCaP cells under 6Gy-irradiated conditions following transporter knockdown, analyzed by flow cytometry. Error bars represent SD (*$p < 0.05$).

Supplementary Figure 7. Targeting AATs alter intracellular ROS and mitochondrial superoxide levels. (A) The Extracellular acidification rate (ECAR) was measured using a Seahorse XF Analyzer in DU145 and LNCaP cells. Error bars represent SEM. **(B)** The ratio of basal oxygen consumption rate (OCR) to basal ECAR in DU145 and LNCaP cells in response to knockdown of *SLC1A5*, *SLC7A5*, and *SLC38A1*. The OCR/ECAR ratio is a metabolic parameter reflecting the balance between mitochondrial respiration and glycolysis. Error bars represent SD. **(C)** Differential expression of genes playing role in Mitochondrial Energy production and metabolism in LNCaP vs. DU145 cells. **(D)** Effect of the *SLC1A5*, *SLC7A5*, *SLC38A1* knockdown on the expression of genes playing role in Mitochondrial Energy Metabolism in DU145 and LNCaP cells. Asterisks represent the statistically significant changes. **(E)** The geneset attributed to KEGG oxidative phosphorylation is upregulated, and the geneset consisting of the Reactome Glycolysis pathway is downregulated by SLC38A1 knockdown in LNCaP cells compared to DU145 cells. The Kruskal-Wallis test was used for statistical analysis followed by post-hoc Dunn's test (*$p < 0.05$). **(F)** Reactive oxygen species (ROS) levels in DU145 and LNCaP cells measured by CM-H2DCFDA staining following transporter knockdown under sham (0 Gy) and irradiated (6 Gy) conditions. Error bars represent SD (*$p < 0.05$, **$p<0.01$).

Supplementary Figure 8. Validation of stable shRNA-mediated knockdown of glutamine transporters in DU145 and LNCaP cells. (A) Western blot analysis confirming the stable knockdown of SLC1A5, SLC7A5, and SLC38A1 in DU145 cells and LNCaP selected with 5 μg/mL puromycin. GAPDH was used as a loading control. **(B)** qRT-PCR analysis confirming the stable knockdown of *SLC1A5*, *SLC7A5*, and

SLC38A1 in DU145 and LNCaP cells selected with 5 µg/mL puromycin. Expression levels are normalized to *ACTB* and *RPLP0*, and presented as fold change relative to cells transfected with control shRNA vector.

Supplementary Figure 9. The interplay between SLC genes and NUPR1 expression and transcriptional activity in prostate cancer cells. (A) qRT-PCR analysis of *SLC1A5*, *SLC7A5*, *SLC38A1*, and *NUPR1* expression in DU145 and LNCaP cells upon siRNA-mediated knockdown of *NUPR1*. Error bars represent SD (*p < 0.05, **p < 0.01, ****p < 0.0001). **(B)** Pearson correlation of mRNA expression levels of *SLC1A5*, *SLC7A5*, and *SLC38A1* genes in the TCGA PRAD patient cohort (n = 498). **(C)** A top 20 enriched Gene Ontology (GO) terms in the set of the NUPR1 target genes (n = 43). The statistical data for GO analysis is provided in Supplementary Table 9. **(D)** A pathway-enrichment analysis of *NUPR1*-responsive genes that are up- or downregulated in response to the *SLC1A5*, *SLC7A5*, or *SLC38A1* knockdown in DU145 and LNCaP cell lines as shown in Figure 5G.

Supplementary Figure 10. Depletion of *NUPR1* alters cell cycle progression, and ROS levels. (A) Cell cycle distribution of DU145 cells under sham-irradiated conditions or after irradiation with 6 Gy following siRNA-mediated knockdown of *NUPR1*. Error bars represent SD (*p < 0.05, **p < 0.01). **(B)** Cell cycle distribution of LNCaP cells under sham-irradiated conditions or after irradiation with 6 Gy following siRNA-mediated knockdown of *NUPR1*. Error bars represent SD (*p < 0.05). **(C)** ROS levels in DU145 and LNCaP cells measured by CM-H2DCFDA staining following *NUPR1* knockdown under sham and irradiated conditions. Error bars represent SD (*p<0.05, **p < 0.01).

Supplementary Figure 11. Effects of *NUPR1* depletion on cancer stem cell properties, cell death, and DNA damage in prostate cancer cells. (A) Bar chart showing plating efficiency (%) of DU145 and LNCaP cells upon *NUPR1* depletion and ZZW-115 treatment. Error bars represent SD. **(B)** DU145, and LNCaP were treated for

48 h with different doses of *NUPR1* inhibitor ZZW-115 at concentrations ranging from 100 µM to 0 µM and viability calculated via Cell-Titer Glo assay. IC_{50} values (50% lethal dose) were determined for each cell line individually by non-linear regression analysis using GraphPad Prism software (San Diego, USA). Error bars represent SD. **(C)** Bar chart showing plating efficiency (%) of DU145 and LNCaP cells upon ZZW-115 treatment. Error bars represent SD. **(D)** qRT-PCR analysis of the indicated gene expression in DU145 and LNCaP cells upon siRNA-mediated knockdown of *NUPR1* ± 6Gy irradiation. Error bars represent SD (*$p < 0.05$, **$p < 0.01$, ***$p < 0.001$). **(E)** Analysis of the *NUPR1* correlation with RT2 gene sets using the publicly available PCa dataset (PRAD) from The Cancer Genome Atlas (TCGA) (n = 498).

Supplementary references:

1. Hevler JF, Zenezeni Chiozzi R, Cabrera-Orefice A, Brandt U, Arnold S, Heck AJR: **Molecular characterization of a complex of apoptosis-inducing factor 1 with cytochrome c oxidase of the mitochondrial respiratory chain**. *Proc Natl Acad Sci U S A* 2021, **118**(39).
2. Sayyed UMH, Mahalakshmi R: **Mitochondrial protein translocation machinery: From TOM structural biogenesis to functional regulation**. *J Biol Chem* 2022, **298**(5):101870.
3. Sevrioukova IF: **Apoptosis-inducing factor: structure, function, and redox regulation**. *Antioxid Redox Signal* 2011, **14**(12):2545-2579.
4. Susin SA, Lorenzo HK, Zamzami N, Marzo I, Snow BE, Brothers GM, Mangion J, Jacotot E, Costantini P, Loeffler M *et al*: **Molecular characterization of mitochondrial apoptosis-inducing factor**. *Nature* 1999, **397**(6718):441-446.
5. Wischhof L, Scifo E, Ehninger D, Bano D: **AIFM1 beyond cell death: An overview of this OXPHOS-inducing factor in mitochondrial diseases**. *EBioMedicine* 2022, **83**:104231.
6. Rodgers JT, Lerin C, Haas W, Gygi SP, Spiegelman BM, Puigserver P: **Nutrient control of glucose homeostasis through a complex of PGC-1alpha and SIRT1**. *Nature* 2005, **434**(7029):113-118.
7. Schopf FH, Biebl MM, Buchner J: **The HSP90 chaperone machinery**. *Nat Rev Mol Cell Biol* 2017, **18**(6):345-360.
8. Myatt SS, Lam EW: **The emerging roles of forkhead box (Fox) proteins in cancer**. *Nat Rev Cancer* 2007, **7**(11):847-859.
9. Radhakrishnan SK, Gartel AL: **FOXM1: the Achilles' heel of cancer?** *Nat Rev Cancer* 2008, **8**(3):c1; author reply c2.
10. Dai C, Sampson SB: **HSF1: Guardian of Proteostasis in Cancer**. *Trends Cell Biol* 2016, **26**(1):17-28.
11. Liu L, Zhao Q, Xiong D, Li D, Du J, Huang Y, Yang Y, Chen R: **Suppressing mitochondrial inner membrane protein (IMMT) inhibits the proliferation of breast cancer cells through mitochondrial remodeling and metabolic regulation**. *Sci Rep* 2024, **14**(1):12766.
12. John GB, Shang Y, Li L, Renken C, Mannella CA, Selker JM, Rangell L, Bennett MJ, Zha J: **The mitochondrial inner membrane protein mitofilin controls cristae morphology**. *Mol Biol Cell* 2005, **16**(3):1543-1554.
13. Katiyar A, Fujimoto M, Tan K, Kurashima A, Srivastava P, Okada M, Takii R, Nakai A: **HSF1 is required for induction of mitochondrial chaperones during the mitochondrial unfolded protein response**. *FEBS Open Bio* 2020, **10**(6):1135-1148.
14. Park HJ, Carr JR, Wang Z, Nogueira V, Hay N, Tyner AL, Lau LF, Costa RH, Raychaudhuri P: **FoxM1, a critical regulator of oxidative stress during oncogenesis**. *EMBO J* 2009, **28**(19):2908-2918.
15. Black M, Arumugam P, Shukla S, Pradhan A, Ustiyan V, Milewski D, Kalinichenko VV, Kalin TV: **FOXM1 nuclear transcription factor translocates into mitochondria and inhibits oxidative phosphorylation**. *Mol Biol Cell* 2020, **31**(13):1411-1424.

Supplementary Table 1. The siRNA sequences used in the study.

siRNAs	siRNA ID	Target sequence (5'-3')
Scr Pool	ScrambledMCR	GCAGCUAUAUGAAUGUUGU
	NonSpecific68%GC	UGCGCUAGGCCUCGGUUGC
	Scr EGFP	GGCUACGUCCAGGAGCGCA
SLC7A5 Pool	SLC7A5 #1	GAGGAUGGAAUUACUUGAA
	SLC7A5 #2	GCCUCAAGGAUACAGGGAG
	SLC7A5 #3	AACAGAGACAAGAAAGGCA
SLC1A5 Pool	SLC1A5-1260	GGAAUAUCACCGGAACCAG
	SLC1A5-896	CCUCAAUGUAGAAGGUGAC
	SLC1A5-948	GACCGUACGGAGUCGAGAA
SLC38A1 Pool	SLC38A1-130944	CCGAAGUAGAAAAUGGUCA
	SLC38A1-HSS129873	CAGUGCCCGAGGAUGAUAA
	SLC38A1-s37590	GGAGUUACAUCUGCUAACA
SLC7A8 Pool	SLC7A8-1909	GCGACAUGUACACACUCAU
	SLC7A8-2314	CCACGAAGGACAAGGACGU
	SLC7A8-2212	AGAUGUGUGUGGUCGUGUA
SLC38A5 Pool	SLC38A5-1	CGGGAUAUCUUUGGAGUUA
	SLC38A5-2	CUUCGGAUGCUGUGGGCUA
	SLC38A5-3	GCUACAGGCAAGAACGUGA
	SLC38A5-4	GCCGGUCCAGUUCAUGGAU
SLC7A7 Pool	SLC7A7-161	GGUUGACAGCACUGAGUAU
	SLC7A7-997	CCUAUUAUACUGUGCUAGA
	SLC7A7-416	GGCCCUUUGUUAUGCGGAA
	SLC7A7-1635	GAACAUAAGCGACCGCUUU
SLC7A11 Pool	SLC7A11-5167	ACAUAUCAAUAGAGGGUUA
	SLC7A11-46	GGAACGAGGAGGUGGAGAA
	SLC7A11-3060	AAUGAGAAUCUGUGGAUAA
	SLC7A11-1511	CGAUACAAAUGCCCAGAUA
NUPR1 Pool	NUPR1-253	AGAGGAAACUGGUGACCAA
	NUPR1-473	GAAAGCGCCUCCAACCCUA
	NUPR1-355	CAGCAAUAGAGACGGGACU
	NUPR1-187	GCCGGAAAGGUCGCACCAA
MYC Pool	MYC-23	ACGGAACUCUUGUGCGUAA
	MYC-24	GAACACACAACGUCUUGGA
	MYC-25	AACGUUAGCUUCACCAACA
	MYC-26	CGAUGUUGUUUCUGUGGAA
GLS Pool	GLS-671	GGGUCUGUUACCUAGCUUG
	GLS-720	GGACAAGAGAAAAUACCUG
	GLS-1626	GCAGUUCGAAAUACAUUGA

Supplementary Table 2. The shRNA expression vectors used in the study.

**shRNAs**	**shRNA ID**	**Clone ID**	**Vector Backbone**	**Target Sequences**
shSLC1A5	shSLC1A5-1	TRCN0000043118	pLKO.1	CTGGATTATGAGGAATGGATA
	shSLC1A5-2	TRCN0000043120	pLKO.1	GCCTGAGTTGATACAAGTGAA
shSLC7A5	shSLC7A5-1	TRCN0000043008	pLKO.1	GCATTATACAGCGGCCTCTTT
	shSLC7A5-2	TRCN0000043009	pLKO.1	CTAGATCCCAACTTCTCATTT
shSLC38A1	shSLC38A1-1	TRCN0000043900	pLKO.1	CCTCCTATTGATCTGTTCAAA
	shSLC38A1-2	TRCN0000043901	pLKO.1	CCTGCATTGTTCCAGAGCTAA
shControl	pLKO.1		pLKO.1	CCTAAGGTTAAGTCGCCCTCG

Supplementary Table 3. The primers used in the study.

SLC1A5	SLC1A5-Fw2	CACCATGGTTCTGGTCTCCTG
	SLC1A5-Rv2	GGTGAAGAGGAAGTAGATGAGG
SLC7A5	SLC7A5-Fw	CCTTCATCGCAGTACATCGTGG
	SLC7A5-Rv	GCCTTCACGCTGTAGCAGTTCAC
SLC38A1	SLC38A1-Fw	GCCATTATGGGCAGTGGGAT
	SLC38A1-Rv	ACACCATGCAGCCTGTTTCT
MYC	c-MYC-Fw	CTCCGTCCTCGGATTCTCTGC
	c-MYC-Rv	CTCCAGCAGAAGGTGATCCAG
GLS	GLS1-Fw	GAGGCATTCTACTGGAGATACC
	GLS1-Rev	GCTCCAGCATTTACCATAGG
SLC7A7	SLC7A7-Fw	CGAGCTGCTTCGCCCCTTATG
	SLC7A7-Rv	TGACCGCGATCAGTGCCAATAC
SLC7A8	SLC7A8-Fw	CCACATTTGGAGGAGTTAATGG
	SLC7A8-Rv	GTACATGTCGCTGGTGACC
SLC7A11	SLC7A11-Fw	GCGTGGGCATGTCTCTGAC
	SLC7A11-Rv	GCTGGTAATGGACCAAAGACTTC
SLC38A5	SLC38A5-Fw	CCTGGTTATCGGCACCTTCC
	SLC38A5-Rv	CCAAGTGTTTCATGAGGGCGA
SLC38A7	SLC38A7-Fw	CGACCAGCAGGACAAGATTAT
	SLC38A7-Rv	GATGAAGAGGAAGGCAGTGAG
NUPR1	NUPR1-Fw	GCACCAAGAGAGAAGCTGC
	NUPR1-Rv	GGTCTGGCCTCATCTCCAG
ATF4	ATF4-Fw	CCAACAACAGCAAGGAGGATGC
	ATF4-Rv	GACTAGGGGGGCAAAGAGATCAC
ACTB	ACTB-Fw	ATGGAGTCCTGTGGCATCCA
	ACTB-Rev	AGTACTTGCGCTCAGGAGGA
RPLP0	RPLP0-Fw	CTCAACATCTCCCCCTTCTCCTT
	RPLP0-Rev	TGATGCAACAGTTGGGTAGCC
SOD2	SOD2-Fw	GGTTTTGGGGTATCTGGGCTCCA
	SOD2-Rev	CTTGGCCAACGCCTCCTGGTA
NEK9	NEK9-Fw	GCTGTGATGGGACATTTCTG
	NEK9-Rev	CCAAGGTAAAGGACGTTGTG
E2F7	E2F7-Fw	AGGCAGCCCAGACTAGATTTT
	E2F7-Rev	GCTGGCAGCACTAATGAGCA
SLC2A1	SLC2A1-Fw	CTCCCTGCAGTTTGGCTAC
	SLC2A1-Rev	GGTTCATCATCAGCATTGAATTCC
MTFR2	MTFR2-Fw	CTGAAATGAGCAAACAGAACC
	MTFR2-Rev	CCTCTTATGAATGGGTCTACC
ATF3	ATF3-Fw	GCTGAGGTTTGCCATCCAG
	ATF3-Rv	GTTTCGGCACTTTGCAGCTG
NFE2L2	NFE2L2-Fw	CCATTCCTGAGTTACAGTGTC
	NFE2L2-Rv	GGATGCTGCTGAAGGAATCC
IGF1R	IGF1R-Fw	CCATCAACAATGAGTACAACTACC
	IGF1R-Rv	GCAGAAGTCACGGTCCACAC

SERTAD2	SERTAD2-Fw	CAGGTGGAGCTGCATGTG
	SERTAD2-Rv	CCTGTTTGAGTTCCTCCTG
PLK3	PLK3-Fw	CCTCAAGTACTTGCACCAG
	PLK3-Rv	CAGAGCAGCGTGTACATGAC
BTG1	BTG1-Fw	GGAGCTGCTGGCAGAACAT
	BTG1-Rv	GTGCTGCCTGTCCAATCAGA

Supplementary Table 4. Antibodies used for Western blot analysis.

Antibodies	Catalog Number	Name	Dilution	Company
SLC1A5	#8057	ASCT2 (D7C12) Rabbit mAb	1:1000	Cell Signaling Technology
SLC7A5	sc-374232	LAT1 (D-10)	1:1000	Santa Cruz Biotechnology
SLC38A1	#36057	SNAT1/SLC38A1 (D9L2P) Rabbit mAb	1:1000	Cell Signaling Technology
GAPDH	#sc-25778	GAPDH (FL-335)	1:1000	Santa Cruz Biotechnology
γH2A.X (Ser139)	# 05-636	Anti-phospho-Histone H2A.X (Ser139)	1:1000	Sigma-Aldrich
Anti-Rabbit IgG (Alexa Fluor 488)	#A32731	Anti-Rabbit IgG (Alexa Fluor 488), Donkey	1:350	Thermo Fisher Scientific

Supplementary Table 5. A comparative phosphoproteomics analysis of PCa cell lines DU145 and LNCaP with or without knockdown of Gln AATs.

Uniprot ID	Entrez Gene	Gene Symbol	**log2 DU145 shSCR / DU145 shSLC1A5_1**	**log2 DU145 shSCR / DU145 shSLC7A5_2**	**log2 DU145 shSCR / DU145 shSLC38A1_1**	**pVal**
Q15388	9804	TOMM20	1.115477	0.891851	0.606179	0.004101
A0A6Q8PFE1	9131	AIFM1	0.315573	0.312384	0.396087	0.000238
Q56NI9	157570	ESCO2	0.487969	0.485427	0.393504	0.000126
P07900	3320	HSP90AA1	-0.57167	-0.55439	-0.71209	0.000253
Q96EB6	23411	SIRT1	-0.31171	-0.36355	-0.49228	0.001921

Uniprot ID	Entrez Gene	Gene Symbol	**log2 LNCaP shSCR / LNCaP shSLC1A5_1**	**log2 LNCaP shSCR / LNCaP shSLC7A5_2**	**log2 LNCaP shSCR / LNCaP shSLC38A1_1**	**pVal**
A0A2P9DTZ8	2305	FOXM1	0.329965	0.440997	0.593	0.004101255
Q00613	3297	HSF1	0.310598	0.393149	0.297655	0.000238236
Q08999	5934	RBL2	0.393664	0.590192	0.672692	0.644468847
Q05513	5590	PRKCZ	0.411925	0.311944	0.405679	0.003250252
A0A075B6F9	51070	NOSIP	0.407897	0.686894	0.915098	0.000253379
Q16891	10989	IMMT	0.767339	0.553936	0.67423	0.001921349
Q4LE36	47	ACLY	0.351763	0.453547	0.822654	0.004101255
Q13057	80347	COASY	0.364851	0.332103	0.375552	0.000238236

Supplementary Table 6. The upstream regulators identified in PCa cell lines DU145 and LNCaP using Ingenuity Pathway Analysis (IPA) in response to specified treatments.

DU145 siSLC1A5		DU145 siSLC7A5		DU145 siSLC38A1		LNCaP siSLC1A5		LNCaP siSLC7A5		LNCaP siSLC38A1	
Upstream Regulator	p-value	Upstream Regulator	p-value	Upstream Regulator	p-value	Upstream Regulator	p-value	Upstream Regulator	p-value	Upstream Regulator	p-value
SYVN1	4.12E-08	ERBB2	1.83E-06	NUPR1	1.83E-12	SKP2	5.37E-05	CCND1	5.58E-07	E2F4	6.66E-26
ERBB2	2.68E-06	COPS5	2.45E-06	SUZ12	1.20E-11	FLOT1	6.67E-05	let-7	1.18E-06	NUPR1	4.15E-24
TCF7L2	7.92E-06	GLI1	2.77E-06	ERBB2	7.77E-10	TRPC1	6.80E-05	NUPR1	1.67E-06	ERBB2	1.47E-22
NUPR1	1.62E-05	NUPR1	9.72E-06	CDK4	2.14E-08	PRDM4	8.56E-05	SUZ12	9.40E-06	CCND1	5.06E-18
SUZ12	8.18E-05	SYVN1	1.49E-05	NR3C1	1.09E-07	NUPR1	1.38E-04	CDK4	1.18E-05	RABL6	4.14E-17

DU145 P +/- Gln		DU145 RR +/- Gln		LNCaP P +/- Gln		LNCaP RR +/- Gln	
Upstream Regulator	p-value	Upstream Regulator	p-value	Upstream Regulator	p-value	Upstream Regulator	p-value
NUPR1	7.31E-32	NUPR1	5.61E-24	E2F4	2.98E-31	NUPR1	2.51E-09
TP53	5.52E-20	TP53	1.17E-13	TBX2	1.47E-26	GNE	1.43E-08
TGFB1	6.57E-16	ERBB2	1.82E-12	NUPR1	1.93E-26	CCND1	2.05E-08
ERBB2	1.64E-15	ESR1	1.17E-11	RABL6	3.08E-26	tosedostat	2.25E-07
ESR1	7.65E-14	RABL6	7.09E-11	CCND1	2.65E-25	FOXM1	1.51E-06

Supplementary Figure 1

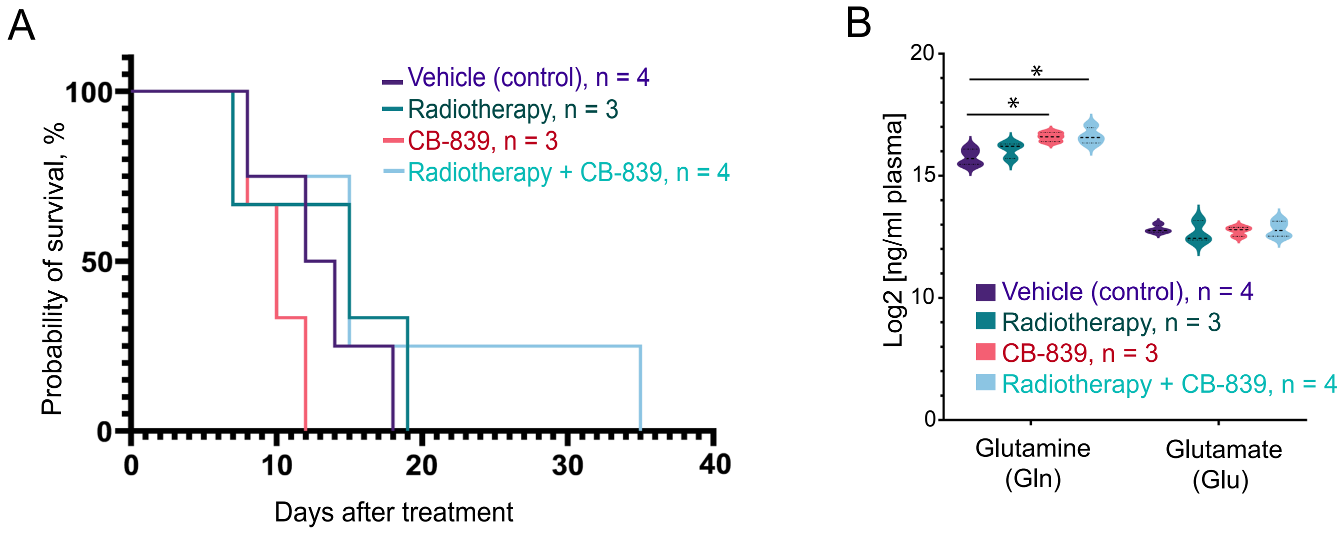

Supplementary Figure 2

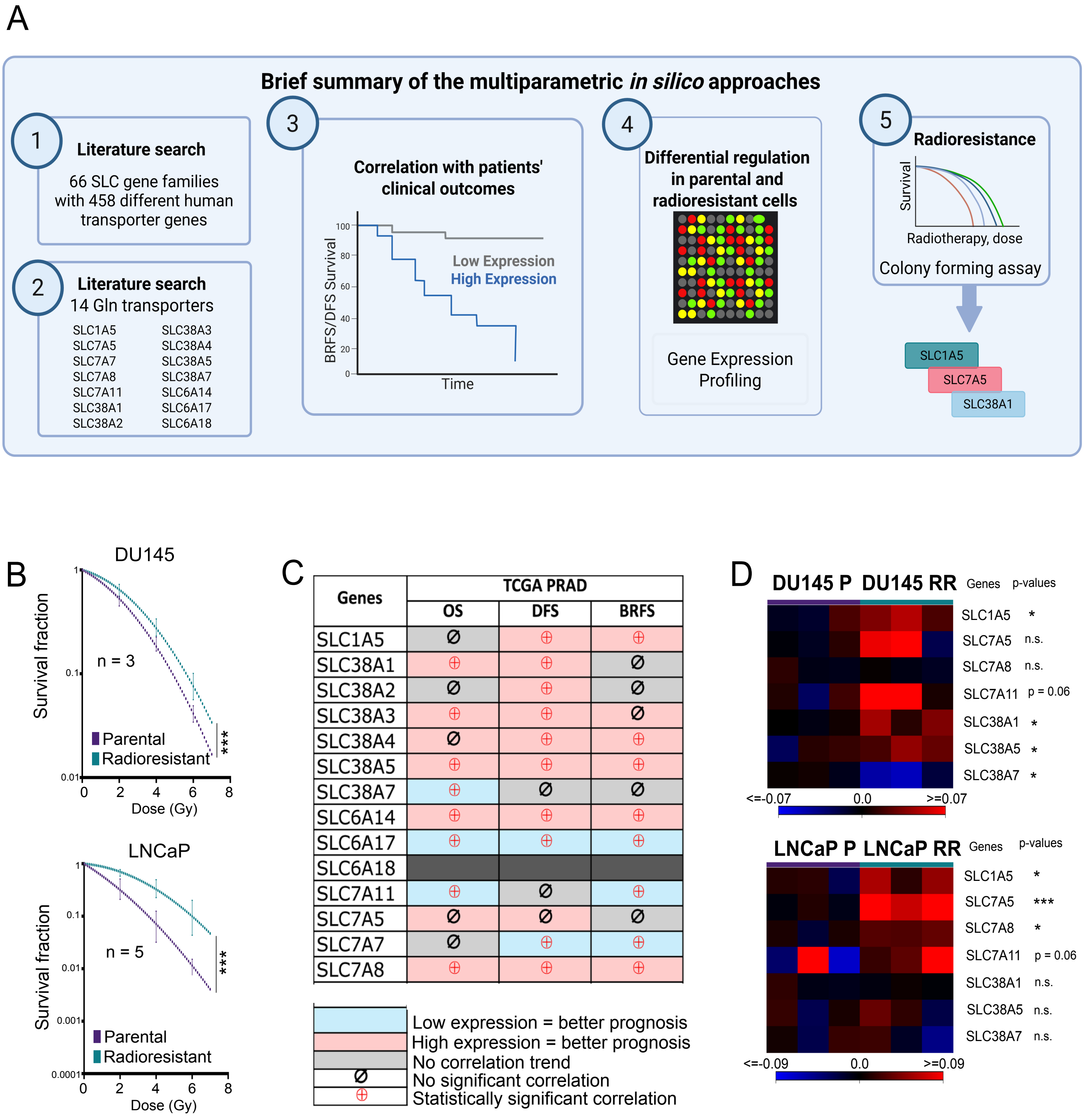

Genes	TCGA PRAD		
	OS	DFS	BRFS
SLC1A5	Ø	⊕	⊕
SLC38A1	⊕	⊕	Ø
SLC38A2	Ø	⊕	Ø
SLC38A3	⊕	⊕	Ø
SLC38A4	Ø	⊕	⊕
SLC38A5	⊕	⊕	⊕
SLC38A7	⊕	Ø	Ø
SLC6A14	⊕	⊕	⊕
SLC6A17	⊕	⊕	⊕
SLC6A18			
SLC7A11	⊕	Ø	⊕
SLC7A5	Ø	Ø	Ø
SLC7A7	Ø	⊕	⊕
SLC7A8	⊕	⊕	⊕

Low expression = better prognosis
High expression = better prognosis
No correlation trend
Ø No significant correlation
⊕ Statistically significant correlation

Supplementary Figure 3

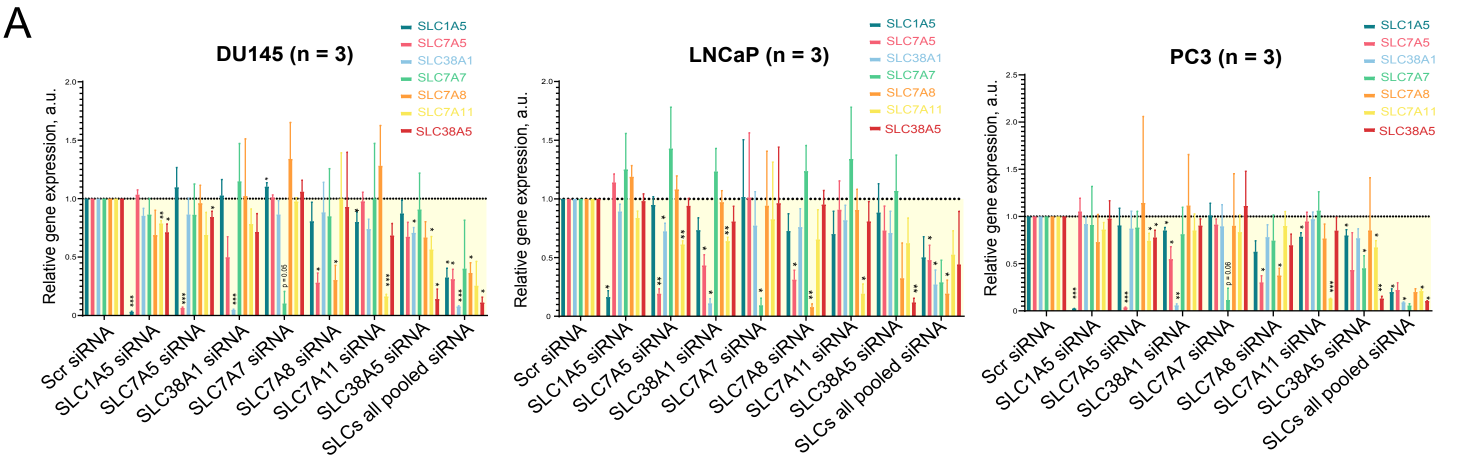

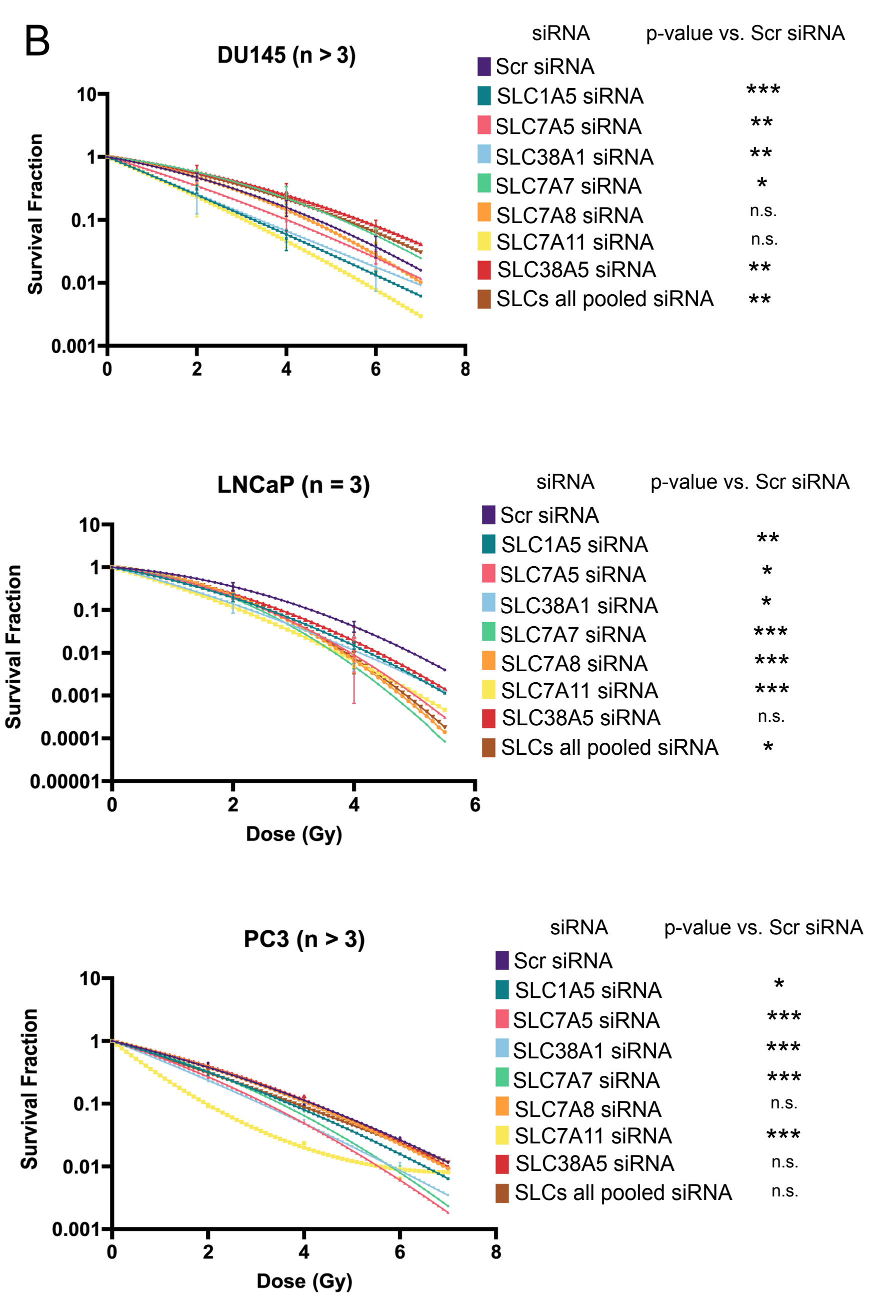

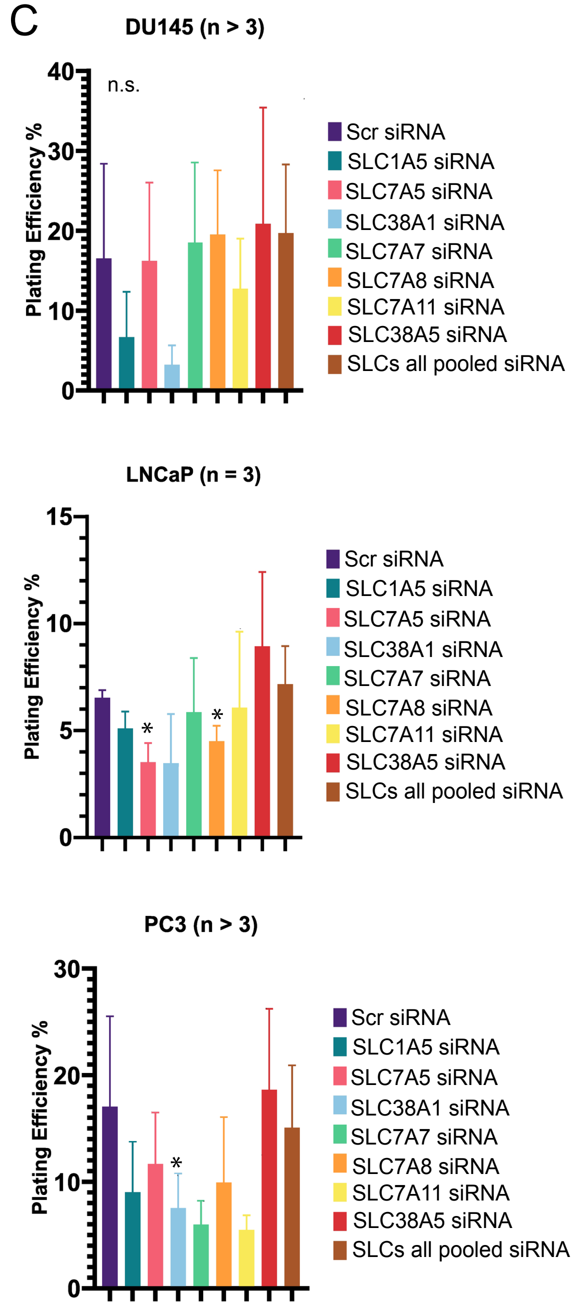

Supplementary Figure 4

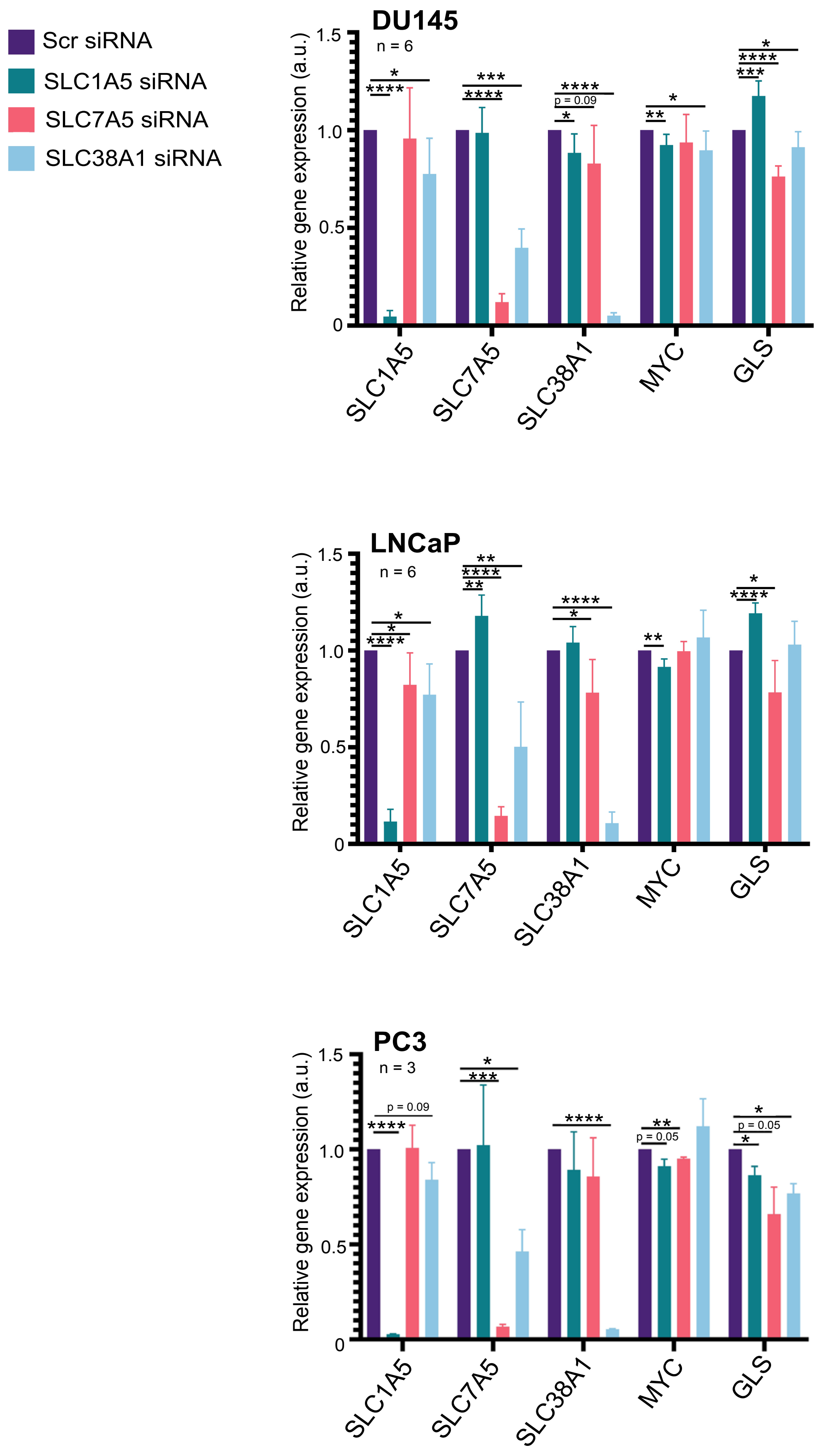

Supplementary Figure 5

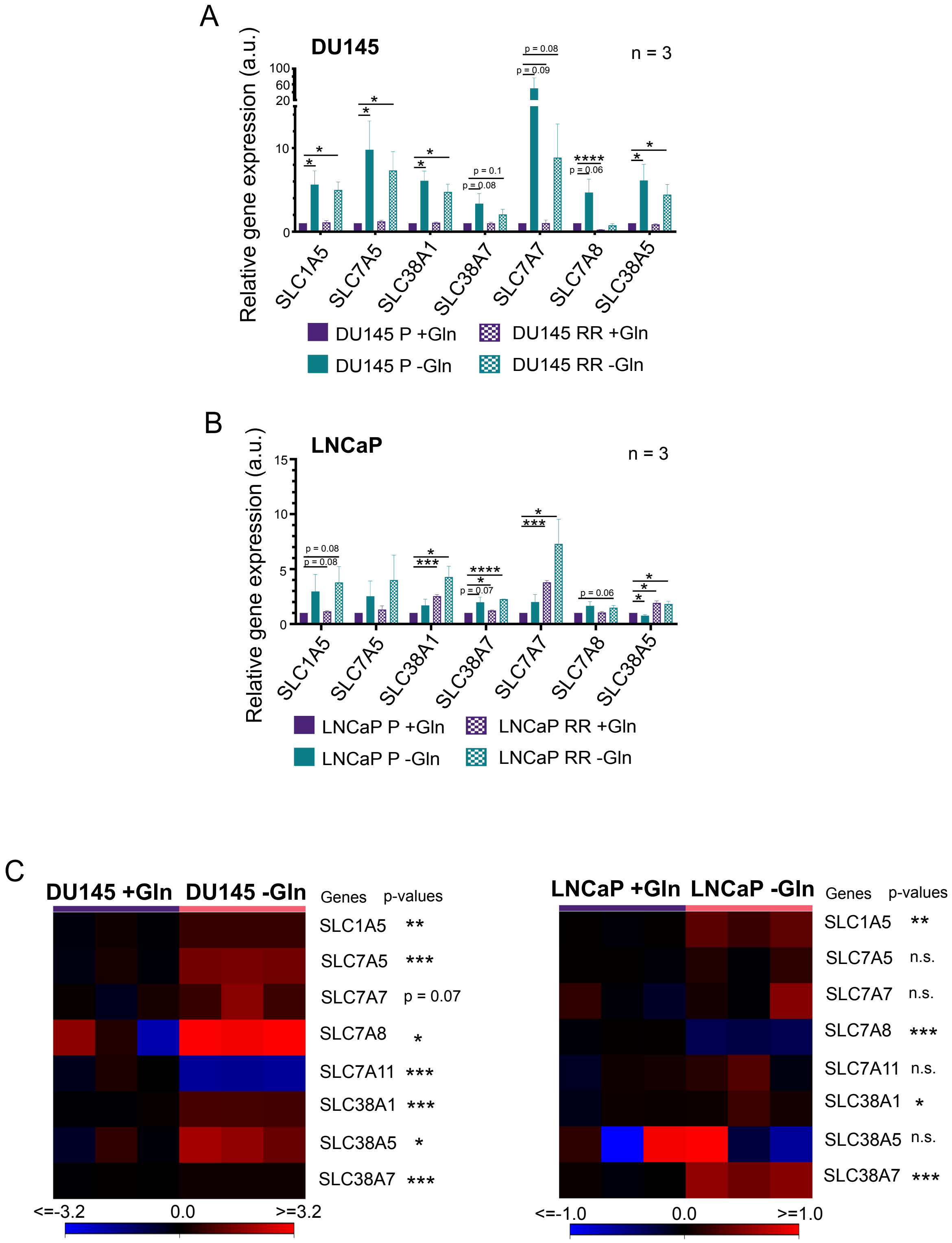

Supplementary Figure 6

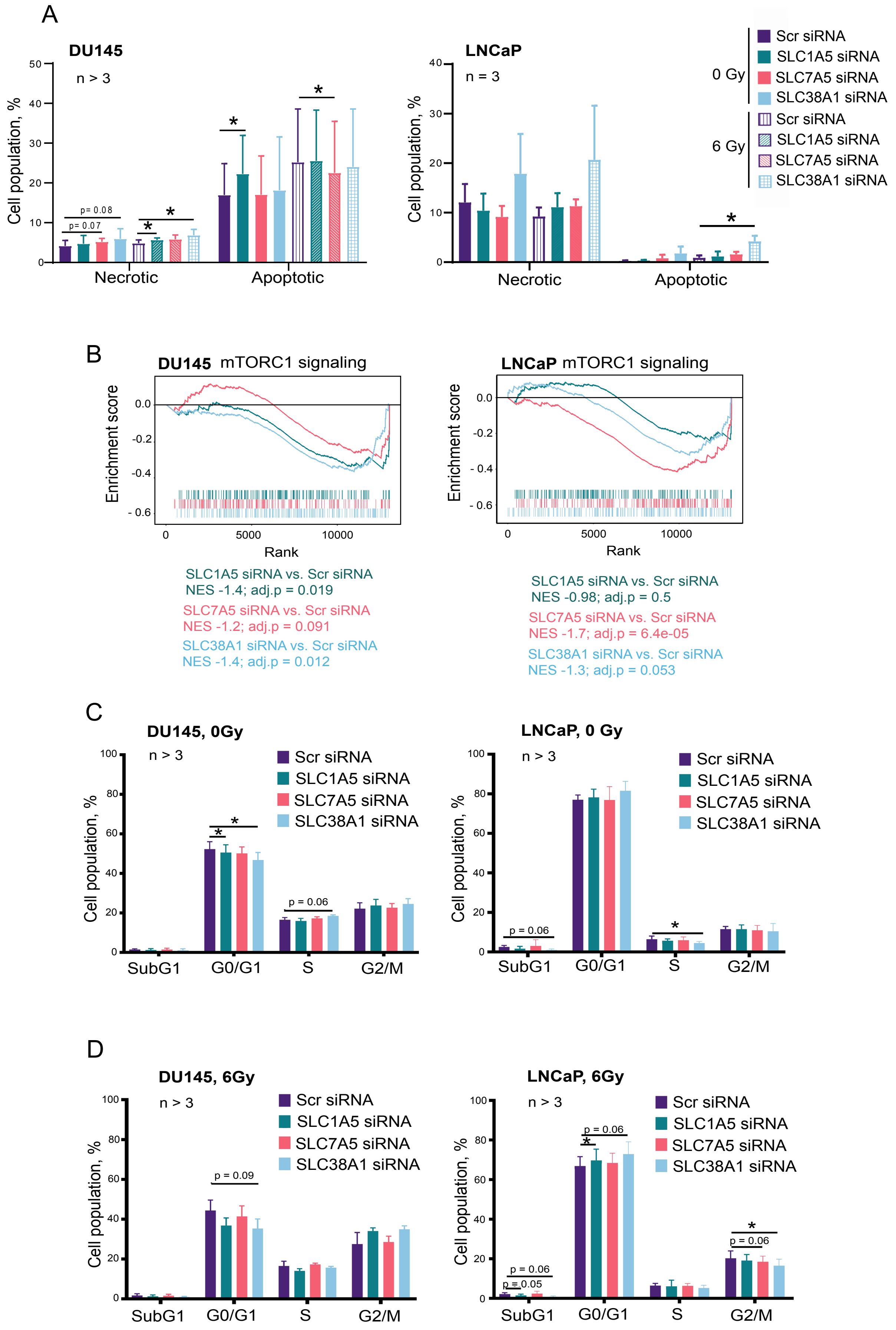

Supplementary Figure 7

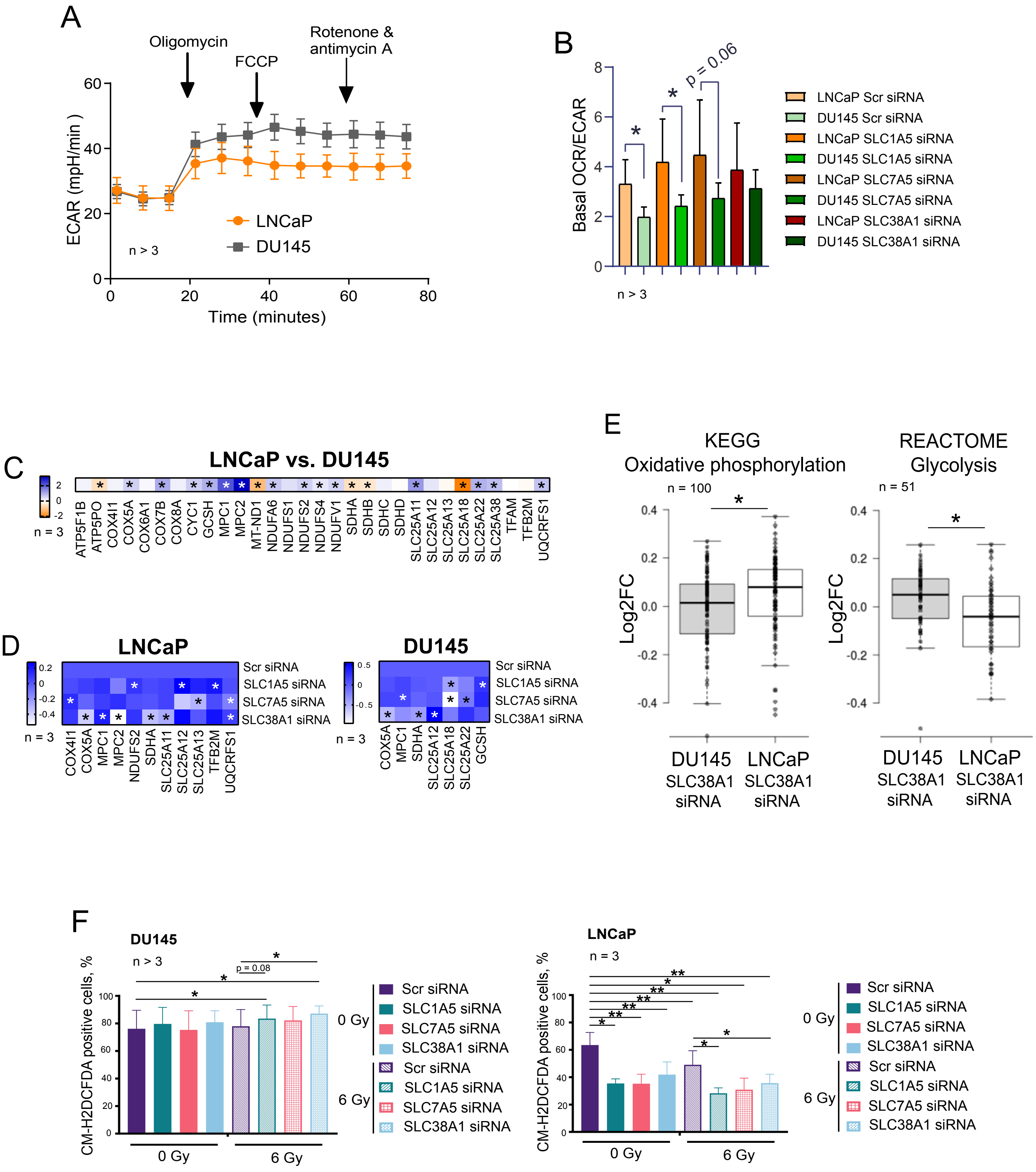

Supplementary Figure 8

A

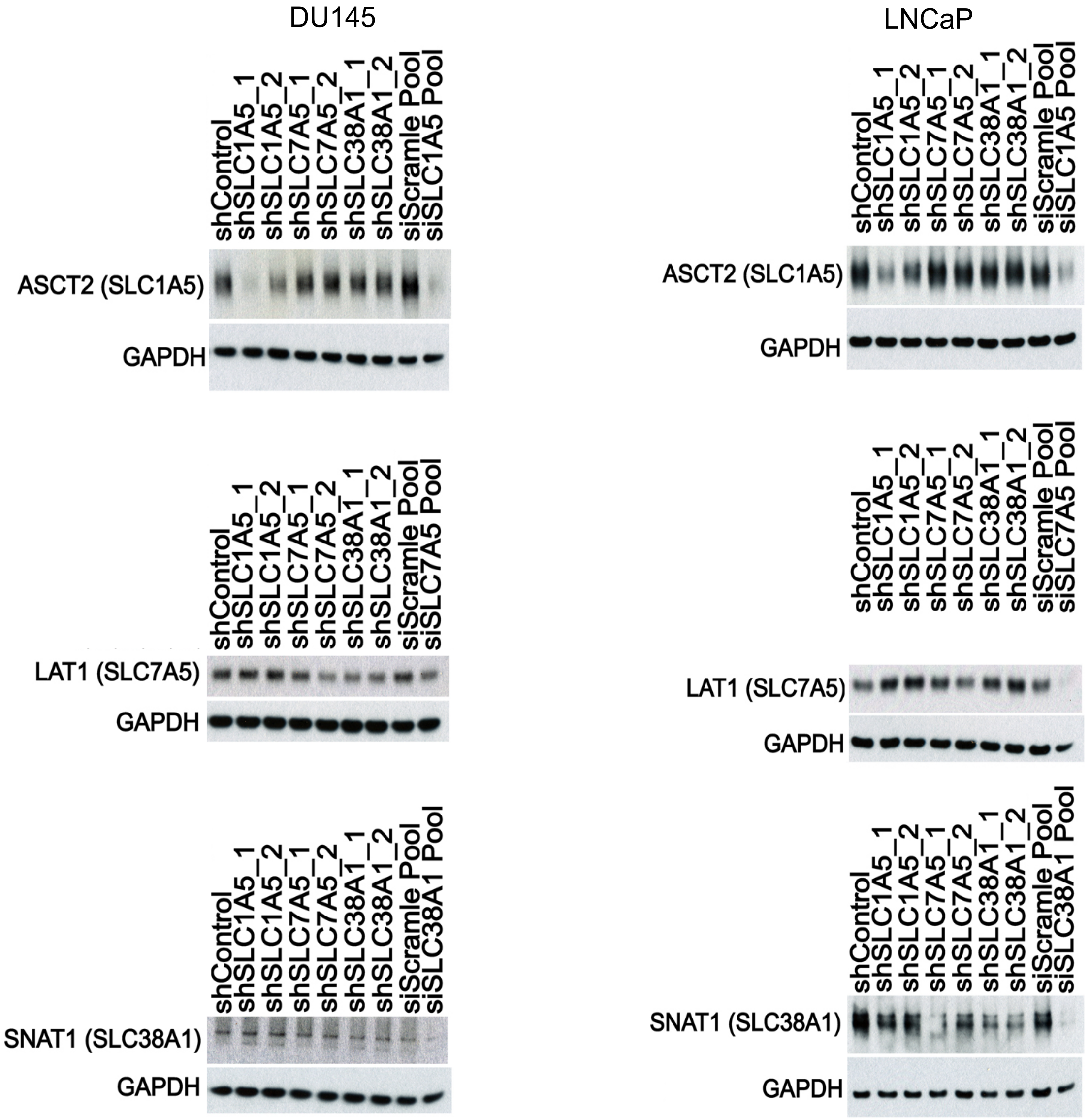

B

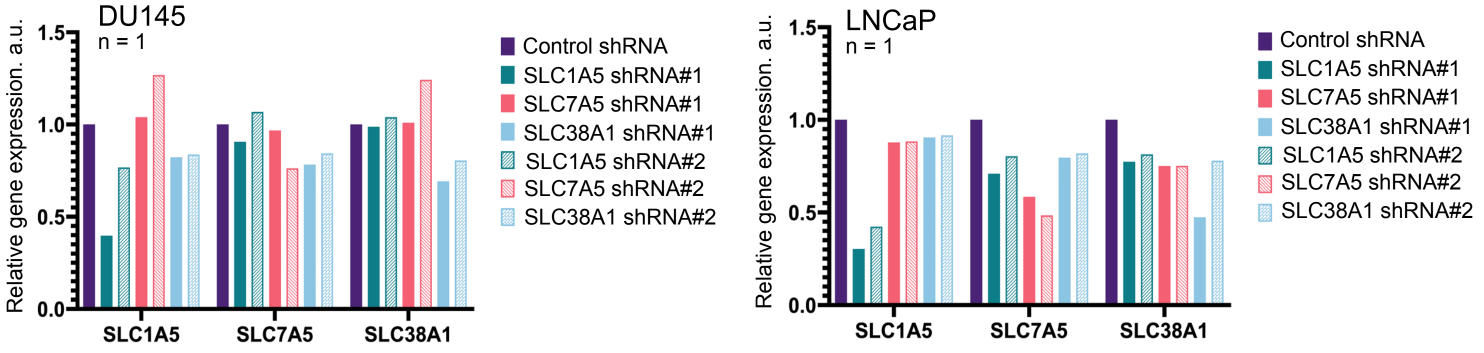

Supplementary Figure 9

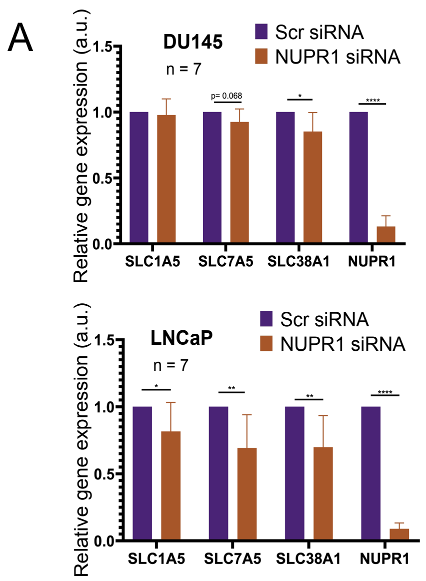

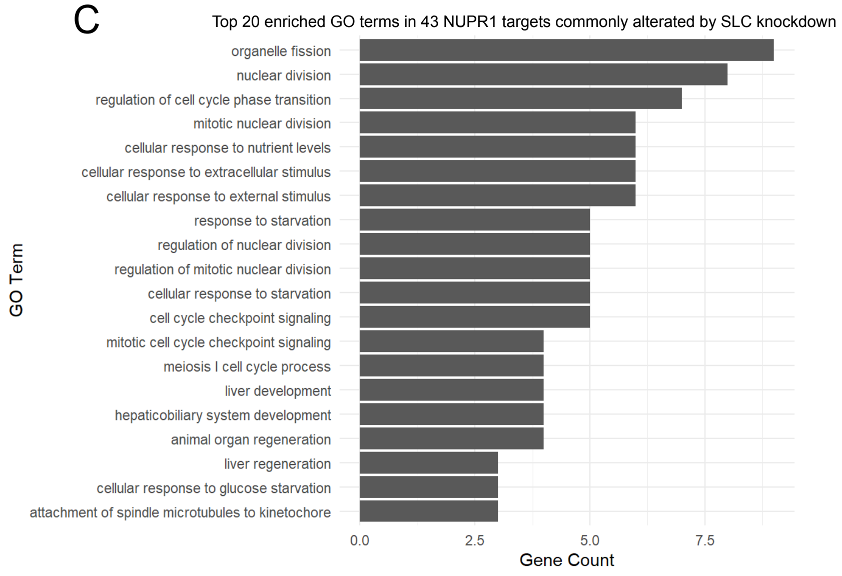

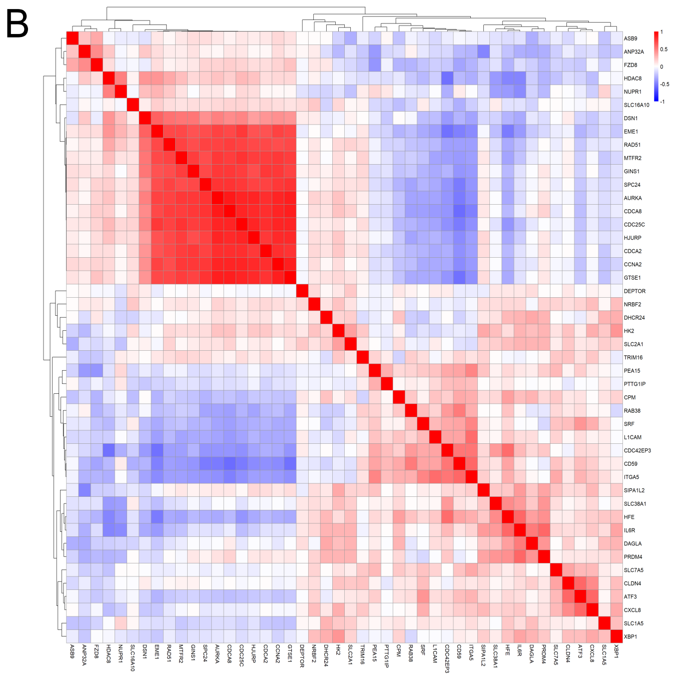

-1 / 1	Correlation Coefficients SLC1A5	SLC7A5	SLC38A1
ANP32A	-0.22292	-0.18701	-0.10811
ASB9	-0.22046	-0.07496	-0.10741
AURKA	-0.12475	-0.03254	-0.01775
CCNA2	-0.23436	-0.02593	-0.01031
CDC25C	-0.17214	-0.05454	-0.11317
CDCA2	-0.20323	-0.00291	-0.0206
CDCA8	-0.13534	-0.09787	-0.00066
DSN1	-0.11246	-0.00656	-0.17931
EME1	-0.13887	-0.06388	-0.25615
MTFR2	-0.20437	-0.02094	-0.11089
FZD8	-0.17041	-0.16774	-0.10636
GINS1	-0.17193	-0.02477	-0.03999
GTSE1	-0.22486	-0.0217	-0.05523
HDAC8	-0.00802	-0.23815	-0.39732
HJURP	-0.11277	-0.06438	-0.03383
RAD51	-0.10356	-0.07176	-0.13544
SPC24	-0.14675	-0.07886	-0.09285

-1 / 1	Correlation Coefficients SLC1A5	SLC7A5	SLC38A1
ATF3	0.026633	0.263693	0.068877
CD59	0.074182	0.2292	0.111082
CDC42EP3	0.071047	0.002763	0.397881
CLDN4	0.077274	0.349355	0.023162
CPM	0.101699	0.215462	0.146849
DAGLA	0.243696	0.018382	0.21403
DEPTOR	0.032157	0.01554	0.081661
DHCR24	0.296877	0.000567	0.109345
HFE	0.207773	0.073539	0.447869
HK2	0.219234	0.10729	0.247335
IL6R	0.130308	0.151446	0.381216
CXCL8	0.088714	0.277026	0.144584
ITGA5	0.13004	0.335074	0.061211
L1CAM	0.063228	0.179785	0.031975
NRBF2	0.025836	0.160357	0.13406
PEA15	0.029267	0.275953	0.056392
PRDM4	0.112486	0.041324	0.412671
PTTG1IP	0.077618	0.005868	0.043694
RAB38	0.036313	0.150404	0.013313
SIPA1L2	0.071387	0.221945	0.204903
SLC16A10	0.032144	0.002314	0.076427
SLC2A1	0.194596	0.062651	0.197205
SRF	0.117095	0.2359	0.032339
TRIM16	0.067784	0.214019	0.016207
XBP1	0.256512	0.050651	0.076273

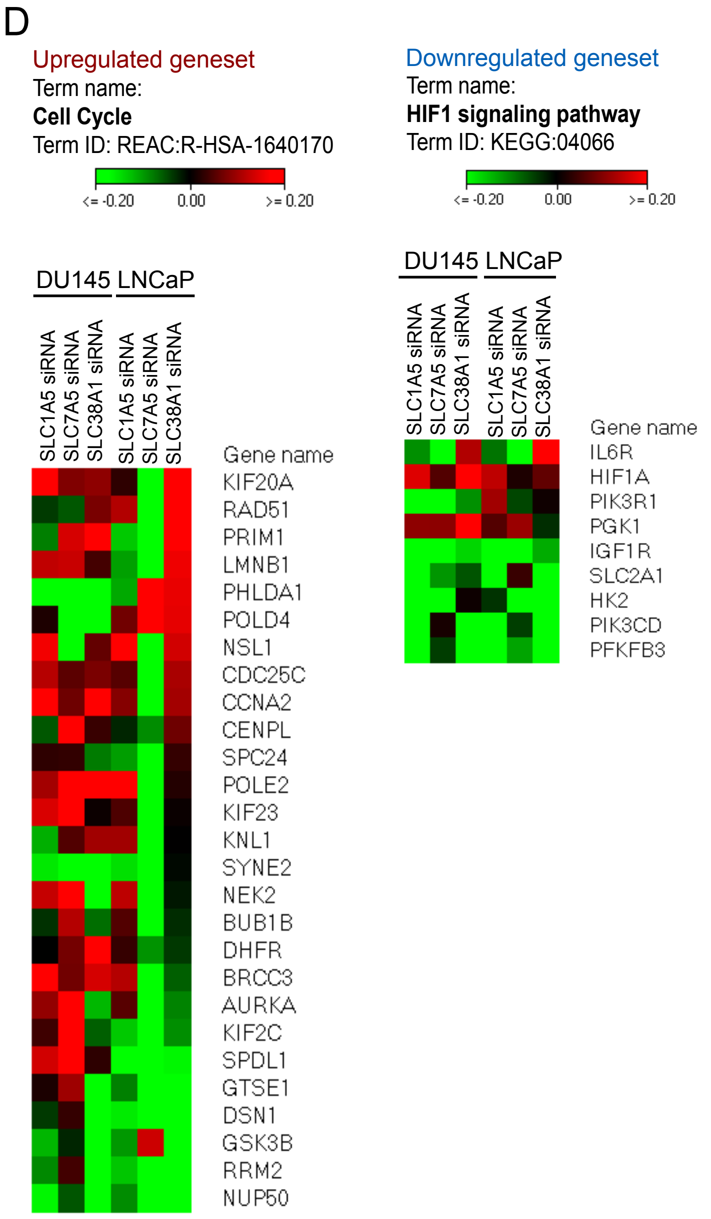

Supplementary Figure 10

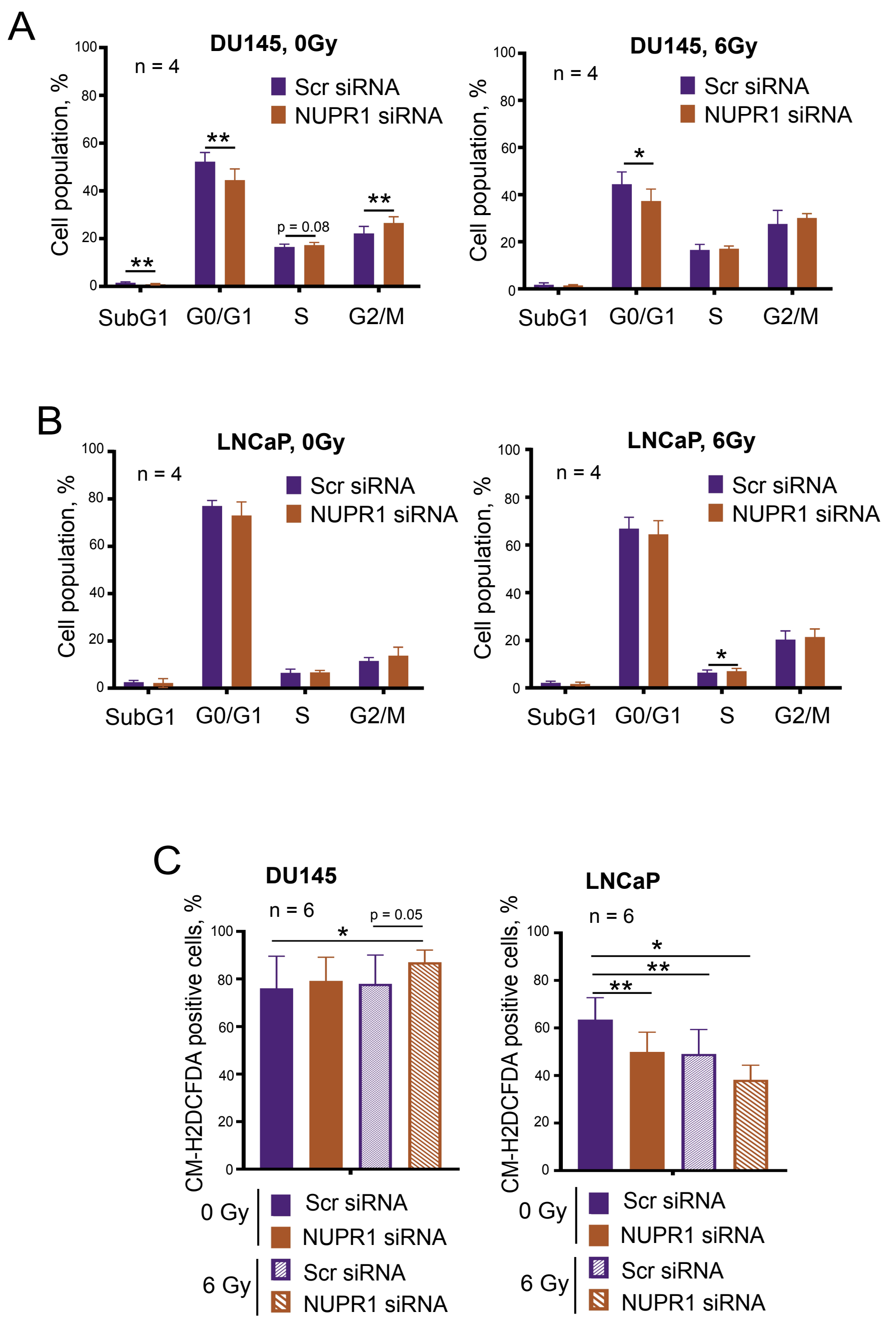

Supplementary Figure 11

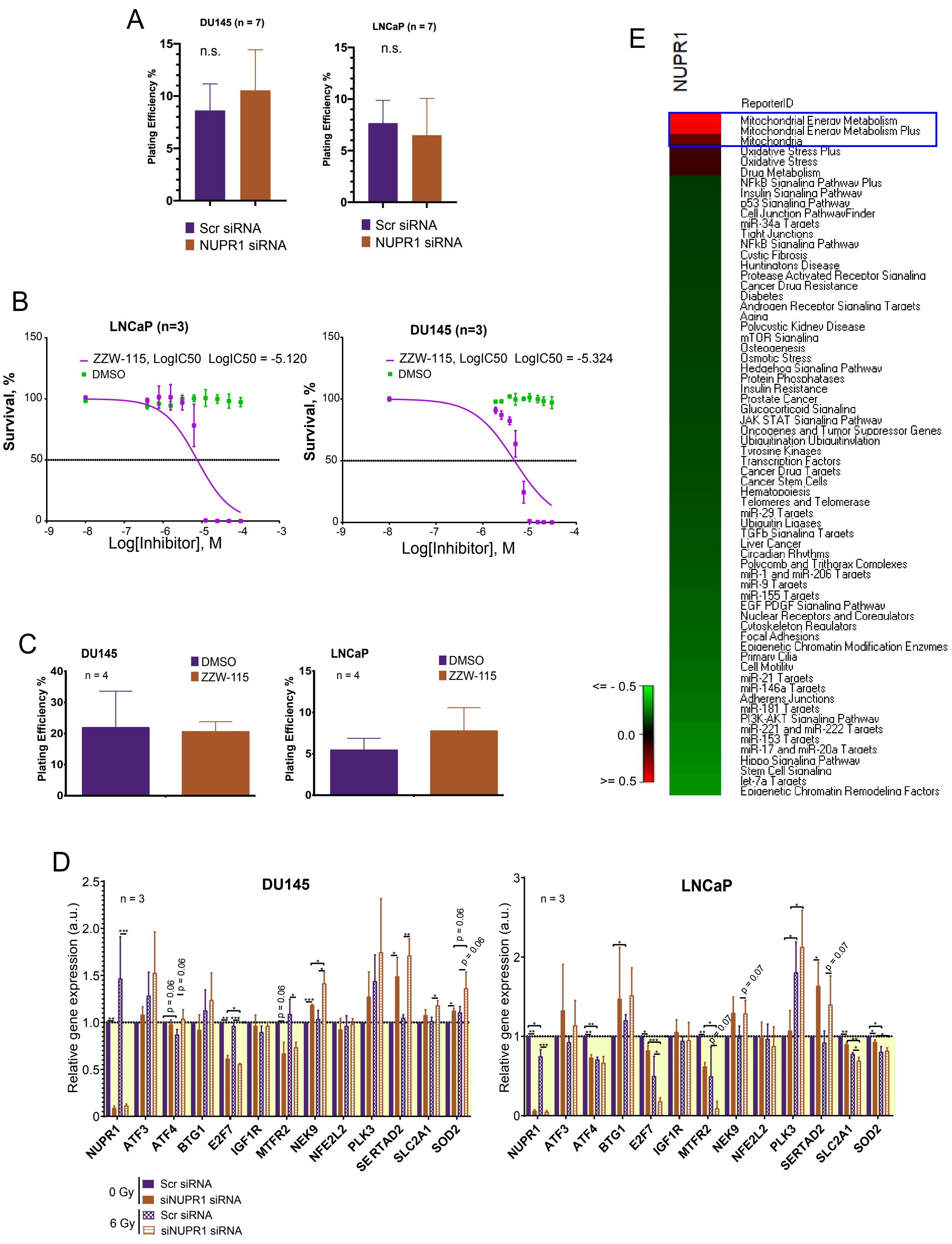

Supplement: Supplementary file 1 — Supplementary Material 1. [file 12964_2025_2344_MOESM1_ESM.zip › Kahya et al Supplementary material_ESM.pdf]
